# Smartphone-Based Artificial Intelligence–Assisted Prediction for Eyelid Measurements: Algorithm Development and Observational Validation Study

**DOI:** 10.2196/32444

**Published:** 2021-10-08

**Authors:** Hung-Chang Chen, Shin-Shi Tzeng, Yen-Chang Hsiao, Ruei-Feng Chen, Erh-Chien Hung, Oscar K Lee

**Affiliations:** 1 Department of Plastic and Reconstructive Surgery Chang Gung Memorial Hospital Taoyuan Taiwan; 2 College of Medicine, Chang Gung University Taoyuan Taiwan; 3 Vendome Plastic Clinic Taipei Taiwan; 4 Institute of Clinical Medicine National Yang Ming Chiao Tung University Taipei Taiwan; 5 Department of Orthopedics China Medical University Hospital Taichung Taiwan

**Keywords:** artificial intelligence, AI, deep learning, margin reflex distance 1, margin reflex distance 2, levator muscle function, smartphone, measurement, eye, prediction, processing, limit, image, algorithm, observational

## Abstract

**Background:**

Margin reflex distance 1 (MRD1), margin reflex distance 2 (MRD2), and levator muscle function (LF) are crucial metrics for ptosis evaluation and management. However, manual measurements of MRD1, MRD2, and LF are time-consuming, subjective, and prone to human error. Smartphone-based artificial intelligence (AI) image processing is a potential solution to overcome these limitations.

**Objective:**

We propose the first smartphone-based AI-assisted image processing algorithm for MRD1, MRD2, and LF measurements.

**Methods:**

This observational study included 822 eyes of 411 volunteers aged over 18 years from August 1, 2020, to April 30, 2021. Six orbital photographs (bilateral primary gaze, up-gaze, and down-gaze) were taken using a smartphone (iPhone 11 Pro Max). The gold-standard measurements and normalized eye photographs were obtained from these orbital photographs and compiled using AI-assisted software to create MRD1, MRD2, and LF models.

**Results:**

The Pearson correlation coefficients between the gold-standard measurements and the predicted values obtained with the MRD1 and MRD2 models were excellent (*r*=0.91 and 0.88, respectively) and that obtained with the LF model was good (*r*=0.73). The intraclass correlation coefficient demonstrated excellent agreement between the gold-standard measurements and the values predicted by the MRD1 and MRD2 models (0.90 and 0.84, respectively), and substantial agreement with the LF model (0.69). The mean absolute errors were 0.35 mm, 0.37 mm, and 1.06 mm for the MRD1, MRD2, and LF models, respectively. The 95% limits of agreement were –0.94 to 0.94 mm for the MRD1 model, –0.92 to 1.03 mm for the MRD2 model, and –0.63 to 2.53 mm for the LF model.

**Conclusions:**

We developed the first smartphone-based AI-assisted image processing algorithm for eyelid measurements. MRD1, MRD2, and LF measures can be taken in a quick, objective, and convenient manner. Furthermore, by using a smartphone, the examiner can check these measurements anywhere and at any time, which facilitates data collection.

## Introduction

Margin reflex distance 1 (MRD1), margin reflex distance 2 (MRD2), and levator muscle function (LF) are crucial for the evaluation and management of ptosis, a condition in which the upper eyelid droops over the eye [1]. MRD1 is defined as the distance between the upper eyelid margin and the center of the pupillary light reflex, whereas MRD2 is defined as the distance between the lower eyelid margin and the center of the pupillary light reflex. The LF is defined as the distance the upper eyelid margin moves from down-gaze to up-gaze without any eyebrow movement. According to a normal MRD1 of 4-5 mm, ptosis can be classified as mild (MRD1: 3-4 mm), moderate (MRD1: 2-3 mm), or severe (MRD1: 0-2 mm).

Manual measurements of MRD1, MRD2, and LF are time-consuming, subjective, and prone to human error [2]. More accurate measurements may be determined using a slit-lamp biomicroscope [3], and several automatic and semiautomatic photographic analysis techniques have been developed to obtain relatively objective measurements of MRD1 and MRD2 [4-6]. However, in these studies, a standardized environment is required for taking the photographs. The Volk Eye Check System measures MRD1 automatically using photographs taken by an integrated camera; however, this system tends to overestimate MRD1 in patients with ptosis [7]. To the best of our knowledge, there are no automatic photographic analysis techniques available for LF measurements.

**Figure 1:**
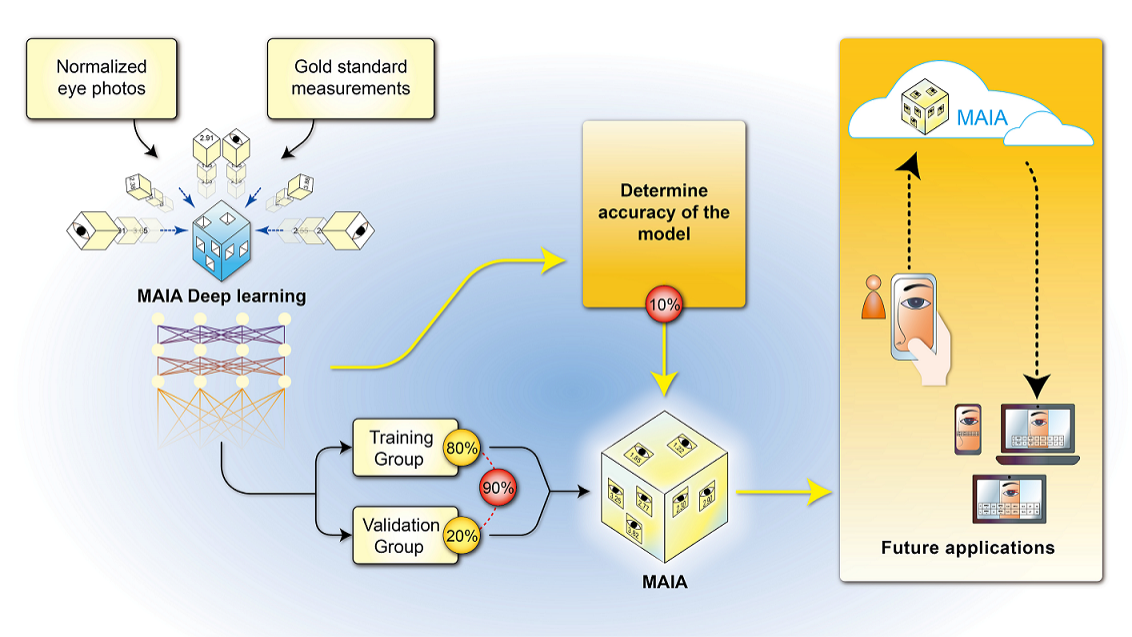
Smartphone-based artificial intelligence–assisted prediction of eyelid measurements. MAIA: medical artificial intelligence assistant (Muen Biomedical and Optoelectronic Technologist, Inc; Version 1.2.0).

A smartphone is more portable and convenient than a traditional photography room and slit-lamp biomicroscope. Artificial intelligence (AI), specifically deep learning (also known as deep neural network learning), is a new and popular area of research that yields impressive results and is growing rapidly. Smartphone-based deep learning image processing is a potential solution to overcome these limitations for measurements of MRD1, MRD2, and LF (Figure 1). We developed the first smartphone-based AI-assisted image processing algorithm for MRD1, MRD2, and LF measurements, which was validated in comparison with gold-standard measurements in an observational study.

## Methods

### Study Design

This observational study included 822 eyes of 411 volunteers aged over 18 years who were referred to a plastic surgery clinic for blepharoplasty between August 1, 2020, and April 30, 2021. The study was approved by the ﻿institutional review board of Chang Gung Memorial Hospital. Volunteers with eyelid defects or deformities, history of corneal injury, enophthalmos, and anophthalmia were excluded.

### Photographs and Gold-Standard Measurements (Actual Values)

A 20×20-mm scale was placed on the nasal dorsum as a reference. The scale was only necessary for gold-standard measurements and was not required for deep learning model training or for determining the accuracy of the model.

Bilateral orbital photographs of each patient (standing or sitting; total 6 photographs including bilateral primary gaze, up-gaze, and down-gaze) were taken using a smartphone (iPhone 11 Pro Max, with flash and a 1:1 ratio) held at the same level between the patient’s eyes at a distance of approximately 20-30 cm, which simulated the distance between the patient and doctor when the doctor uses a handheld ruler to measure MRD1, MRD2, and LF in the clinic.

**Figure 2:**
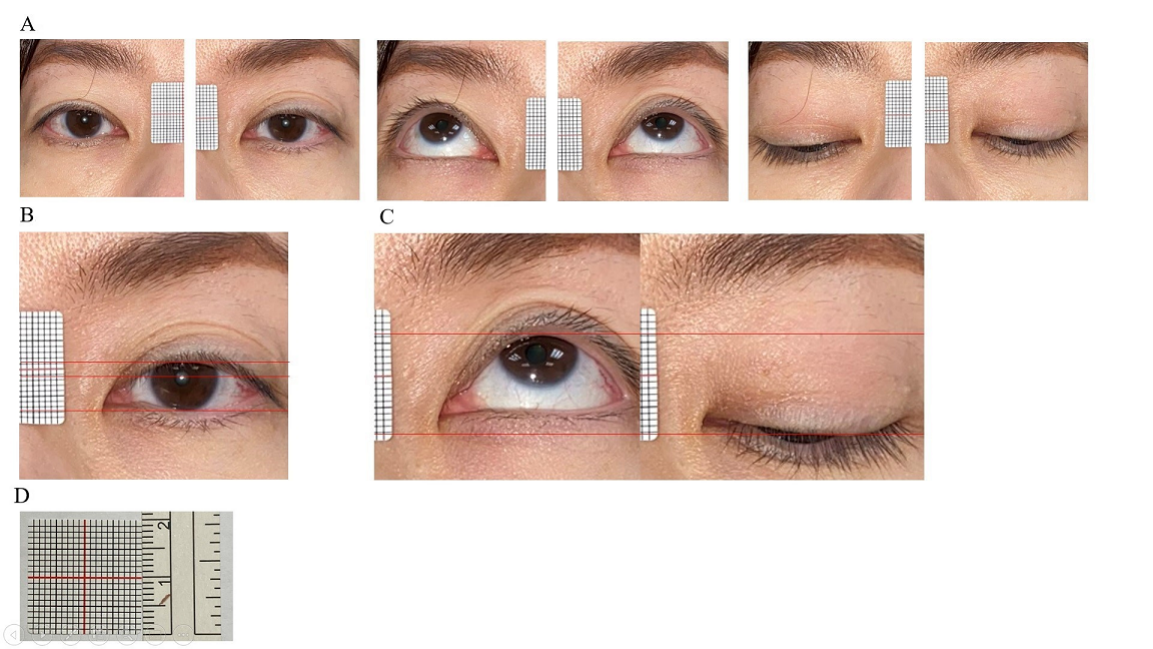
Photographs and gold-standard measurements (real values) (A) Six orbital photographs, including bilateral primary gaze, up-gaze, and down-gaze, were taken by a smartphone. (B) The primary gaze photograph was then magnified for margin reflex distance 1 (MRD1) and MRD2 measurements. (C) The up-gaze and down-gaze photographs were then magnified for levator muscle function (LF) measurements. (D) A 20×20-mm scale.

The photographs were magnified on the computer, and MRD1, MRD2, and LF measurements were taken by two doctors independently (measured in increments of 0.25 mm). The doctors drew a horizontal line across the upper eyelid margin, light reflex, and lower eyelid margin to the 20×20-mm scale to obtain the MRD1, MRD2, and LF measurements. The mean value of measurements obtained by the two doctors was taken as the gold-standard measurement (actual value), which served as the input data for deep learning model training (Figure 2)*.*

Usually, in ptotic eyelids without a corneal light reflex, the distance (in millimeters) that the eyelid must be lifted is recorded as a negative value, which is the MRD1. However, the distance the examiner lifts the eyelid is very subjective and therefore cannot be used as a gold-standard measurement. Accordingly, in this study, all MRD1 measurements in ptotic eyelids without a corneal light reflex were set to 0.

### Photograph Normalization

**Figure 3:**
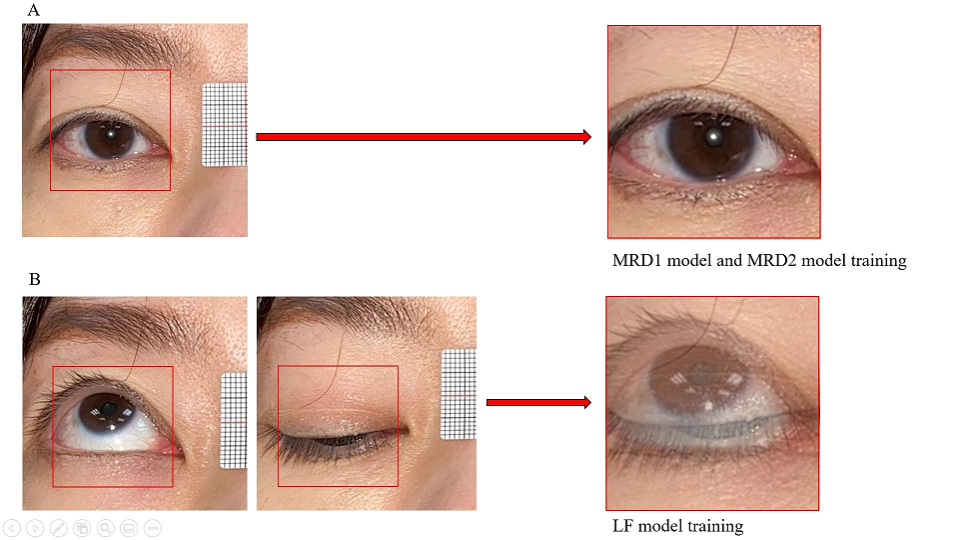
Photograph normalization. (A) Autosegmentation of primary-gaze orbital photographs. These photographs are considered the “normalized eye photographs” for margin reflex distance 1 (MRD1) and MRD2 model training. (B) Autosegmentation of up- and down-gaze orbital photographs, which were then merged into one photograph. These photographs are considered the “normalized eye photographs” for levator muscle function (LF) model training.

Segmentation of primary-gaze orbital photographs (a square region around the light reflex as a center) was automatically performed by our software algorithm. We used LabelImage [8] to label the pupil light reflex location (X, Y), and then built a MobilenetV2 [9] model to train a regression model that can find a pupil light reflex coordinate. Square orbital pictures were automatically cropped (image size/4) using the light reflex coordinate extension after determining the light reflex coordinate. These segmented square photographs were considered the “normalized eye photographs,” which were used as input data for MRD1 and MRD2 deep learning model training. Segmentations of up- and down-gaze orbital photographs were automatically merged into one photograph by our software algorithm. These segmented and merged photographs were considered the “normalized eye photographs” for LF deep learning model training (Figure 3).

### Model Training: Image Analysis by Automatic Deep Learning Software

The normalized eye photographs and gold-standard measurements of MRD1, MRD2, and LF were compiled using medical artificial intelligence assistant (MAIA) software (Muen Biomedical and Optoelectronic Technologist Inc; Version 1.2.0) to analyze the image features and classify different situations. MAIA software automatically optimizes parameters for training models, including multiple convolutional neural network (CNN) models such as SE ResNet and EfficientNet [10,11].

The input data were processed with the following steps: (1) images were resized into 256×256 using a bilinear interpolation method, (2) images were augmented using horizontal flip and randomly rotated using the albumentations method [12], and (3) five-fold cross-validation was used to estimate the performance of the models.

**Figure 4:**
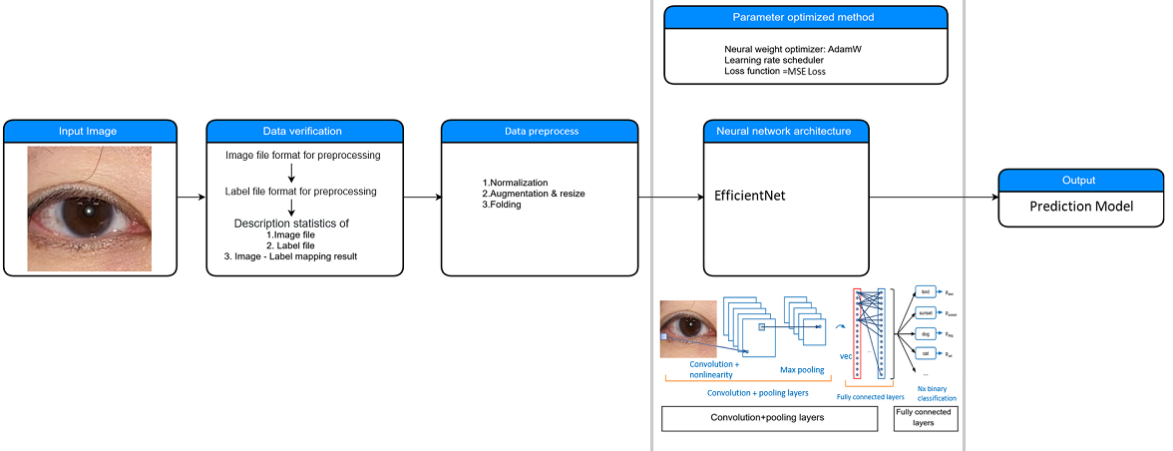
The convolutional neural network architecture of medical artificial intelligence assistant (MAIA) software.

The neural network architecture chooses an optimal network for memory consumption. We added the dropout function and applied different data augmentation methods to prevent the model from overfitting to our dataset [13,14]. The dropout rate was set from 0.25 to 0.5 for regularization. We then trained the model using minibatches of size 32, which were selected based on memory consumption [15]. The learning rate was tuned based on cosine annealing and a one-cycle policy strategy [16,17]. Using the cosine annealing schedule, the model repeatedly fits the gradient to the local minimum. The network was trained end-to-end using the Adam optimization algorithm, which optimized the mean square error as a loss function [18]. Lastly, we ensembled all of the models using the average output of the model to obtain a more robust result, minimize the bias of prediction error, and improve the prediction accuracy of the CNN models (Figure 4).

MAIA software was used with Python 3.x and PyTorch 1.1.x for Windows 10.

### Model Performance Evaluation

In total, three AI models, the MRD1, MRD2, and LF models, were trained. The photograph processing time for each model was recorded. The mean absolute error (MAE) and mean square error (MSE) were selected to evaluate the performance of model prediction. The Pearson correlation coefficient was used to assess the correlation between the deep learning model prediction and gold-standard measurements. The intraclass correlation coefficient (ICC) was used to compare the agreement between the deep learning model prediction and the gold-standard measurements. Statistical analyses were performed using R software (version 4.1.0; R Foundation). Bland-Altman analysis was used to compare the agreement between the deep learning model prediction and the gold-standard measurements. Statistical significance was set at *P*<.05.

## Results

### Data Characteristics

**Figure 5:**
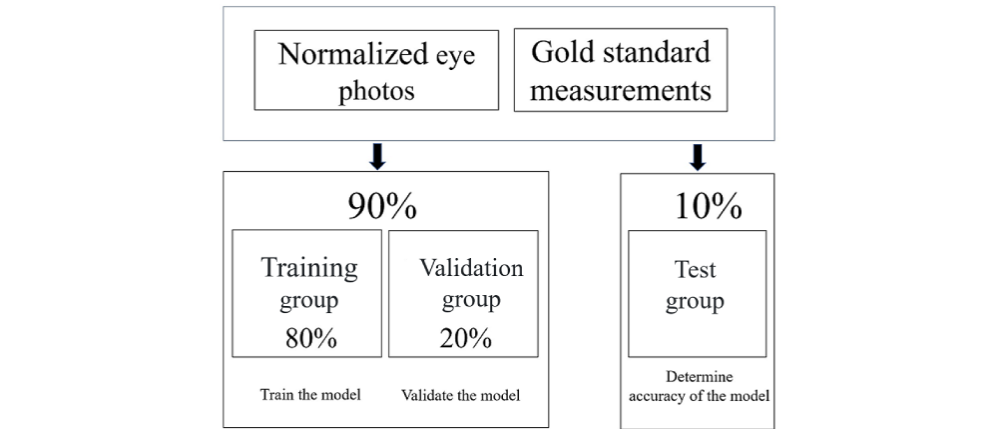
Data organization for model evaluation. Ninety percent of the data were used as the training/validation group and 10% were used as the test group; 80% of the data from the training/validation group were used as the training group and 20% were used as the validation group.

We collected 822 eye photographs from 411 volunteers, including 344 (83.7%) women and 67 (16.3%) men. The photographs were subsequently randomly divided into two groups: 90% as the training/validation group and 10% as the test group. Within the training/validation group, 80% of photographs were used as the training group and 20% were used as the validation group (Figure 5). The case numbers and sex ratios in the MRD1, MRD2, and LF models are shown in Table 1. In the LF model, 137 normalized eye photographs were excluded because the up- and down-gaze orbital photographs were not well merged.

**Table 1:** Case numbers and sex ratios in each model.

Model		Cases, n (%)		Males, n (%)
**MRD1^a^**				
	Total	822 (100.0)	154 (18.7)	
	Training group	740 (90.0)	142 (19.2)	
	Test group	82 (10.0)	12 (14.6)	
**MRD2^b^**				
	Total	822 (100.0)	154 (18.7%)	
	Training group	740 (90.0)	142 (9.2%)	
	Test group	82 (10.0)	12 (14.6%)	
**LF^c^**				
	Total	685 (100.0)^d^	122 (17.8)	
	Training group	617 (90.0)	113 (8.3)	
	Test group	68 (10.0)	9 (13.2)	

^a^MRD1: marginal reflex distance 1.

^b^MRD2: marginal reflex distance 2.

^c^LF: levator muscle function.

^d^In the LF model, 137 normalized eye photographs were excluded because the up- and down-gaze orbital photographs were not well merged.

### Reliability of Gold-Standard Measurements

**Figure 6:**
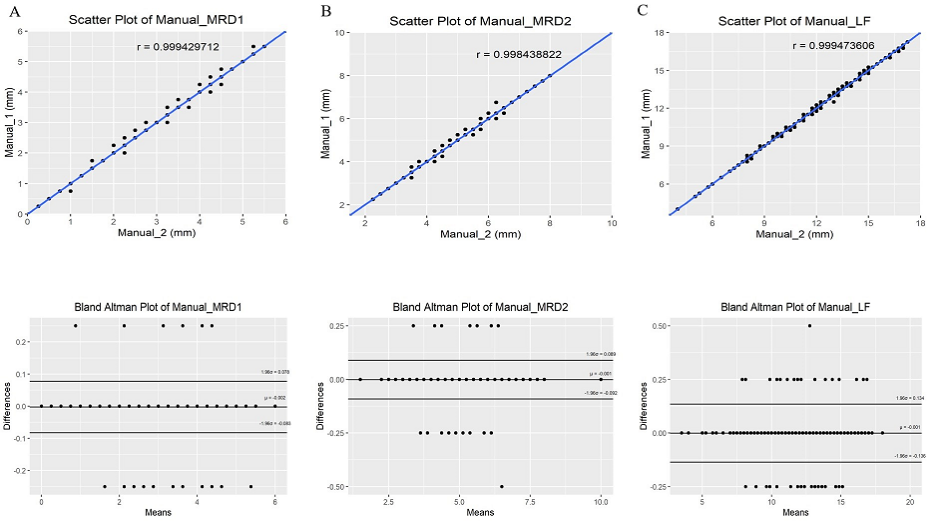
Scatter plots and Bland-Altman plots of gold-standard measurements (real values) for marginal reflex distance (MRD)1 (A), MRD2 (B), and levator muscle function (LF) (C) performed by two doctors.

The gold-standard measurements of MRD1, MRD2, and LF are summarized in Table 2. To determine the reliability, the measurements performed by the two doctors were evaluated using MAE, MSE, Pearson correlation coefficient, ICC, and Bland-Altman analysis. The reliability of the two doctors was excellent (Table 3, Figure 6).

**Table 2:** Summary of gold-standard measurements.

Measurements	N	Mean (SD)	Range
MRD1^a^ (mm)	822	2.59 (1.21)	0.00-6.00
MRD2^b^ (mm)	822	5.51 (0.83)	1.50-10.00
LF^c^ selected (mm)	685^d^	12.1 (2.12)	3.50-18.00

^a^MRD1: marginal reflex distance 1.

^b^MRD2: marginal reflex distance 2.

^c^LF: levator muscle function.

^d^In the LF model, 137 normalized eye photographs were excluded because the up- and down-gaze orbital photographs were not well merged.

**Table 3:** Reliability of gold-standard measurements (actual values) manually performed by the two doctors.

Metric	MRD1^a^	MRD2^b^	LF^c^
MAE^d^	0.007	0.008	0.018
MSE^e^	0.005	0.001	0.002
Pearson correlation coefficient	0.999	0.998	0.999
ICC^f^ (agreement)	0.999	0.998	0.999
ICC (consistency)	0.999	0.998	0.999

^a^MRD1: marginal reflex distance 1.

^b^MRD2: marginal reflex distance 2.

^c^LF: levator muscle function.

^d^MAE: mean absolute error.

^e^MSE: mean square error.

^f^ICC: intraclass correlation coefficient.

### Validation of the Training Model

**Figure 7:**
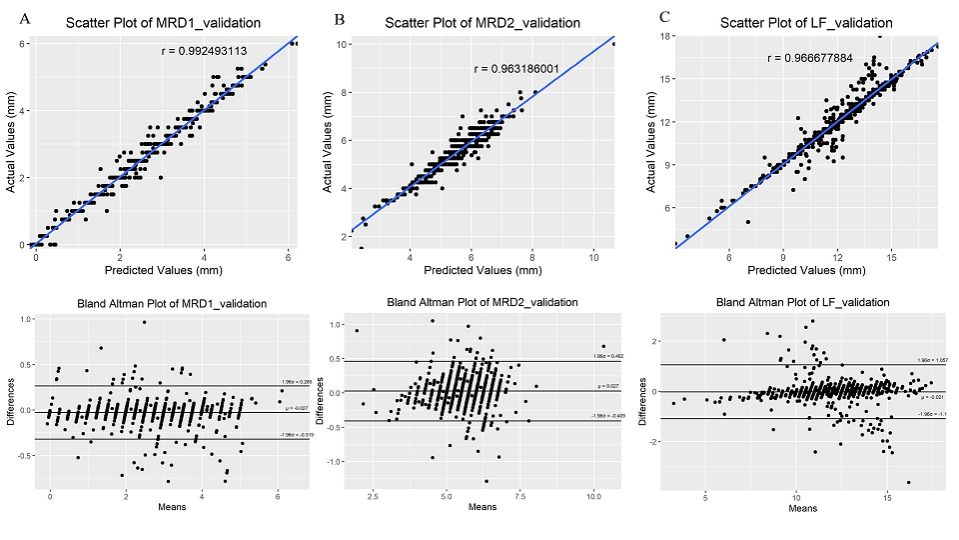
Scatter plots and Bland-Altman plots of validation results of the marginal reflex distance (MRD)1(A), MRD2 (B), and levator muscle function (LF) (C) training models.

There were 740 patients in the training/validation group included in the MRD1 and MRD2 models, and 617 patients included in the training/validation group in the LF model. The validation results based on MAE, MSE, Pearson correlation coefficient, ICC, and Bland-Altman analysis were good overall (Table 4, Figure 7).

**Table 4:** Validation and test results of the training model.

Metric	Validation				Test		
	MRD1^a^	MRD2^b^	LF^c^	MRD1		MRD2	LF
MAE^d^ (mm)	0.087	0.158	0.290	0.349		0.375	1.059
MSE^e^	0.023	0.050	0.303	0.227		0.246	1.709
Pearson correlation coefficient	0.992	0.963	0.967	0.908		0.875	0.728
ICC^f^ (Agreement)	0.992	0.962	0.966	0.903		0.837	0.692
ICC (Consistency)	0.992	0.963	0.966	0.902		0.837	0.689

^a^MRD1: marginal reflex distance 1.

^b^MRD2: marginal reflex distance 2.

^c^LF: levator muscle function.

^d^MAE: mean absolute error.

^e^MSE: mean square error.

^f^ICC: intraclass correlation coefficient.

### Test Results of the MRD1, MRD2, and LF models

A total of 82 patients were used as the test group in the MRD1 and MRD2 models, and 68 patients were used as the test group in the LF model. The test results determine the accuracy of the model. It took 2.09 seconds and 2.15 seconds for the MRD1 and MRD2 models to respectively process 82 photos, and it took 1.97 seconds for the LF model to process 68 photos. The MAE of the predicted values to the gold-standard measurements of MRD1, MRD2, and LF were 0.35 mm, 0.37 mm, and 1.06 mm, respectively, and the MSE of the predicted values to the gold-standard measurements of MRD1, MRD2, and LF were 0.23 mm, 0.25 mm, and 1.71 mm, respectively.

The correlations between the gold-standard measurements and the values predicted by the MRD1 and MRD2 models were excellent (*r*=0.91 and 0.88, respectively). The correlation between the test results obtained with the LF model and gold-standard measurements was good (*r*=0.73).

The ICCs (agreement) between the gold-standard measurements and the values predicted with the MRD1, MRD2, and LF models were 0.90, 0.84, and 0.69, respectively. The ICCs (consistency) between the gold-standard measurements and the values predicted with the MRD1, MRD2, and LF models were 0.90, 0.84, and 0.69, respectively. These results indicate excellent agreement between the gold-standard measurements and the values predicted with the MRD1and MRD2 models, and substantial agreement with the LF model [19].

**Figure 8:**
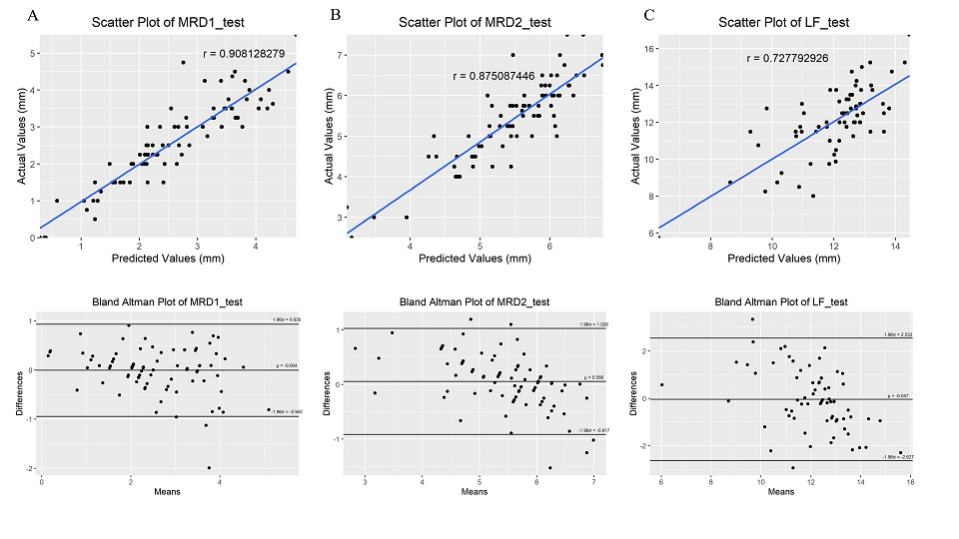
Scatter plots and Bland-Altman plots of the test results of the marginal reflex distance (MRD)1(A), MRD2 (B), and levator muscle function (LF) (C) training models.

Bland-Altman analyses showed that the bias between the gold-standard measurements and the values predicted by the MRD1, MRD2, and LF models was –0.004 mm (95% CI –0.1090 to 0.1015 mm), 0.056 mm (95% CI –0.05347 to 0.1646 mm), and –0.047 mm (95% CI –0.3658 to 0.2713 mm), respectively. The 95% limits of agreement were −0.94 to 0.94 mm for the MRD1 model, –0.92 to 1.03 mm for the MRD2 model, and –2.63 to 2.53 mm for the LF model (Table 4, Figure 8).

**Figure 9:**
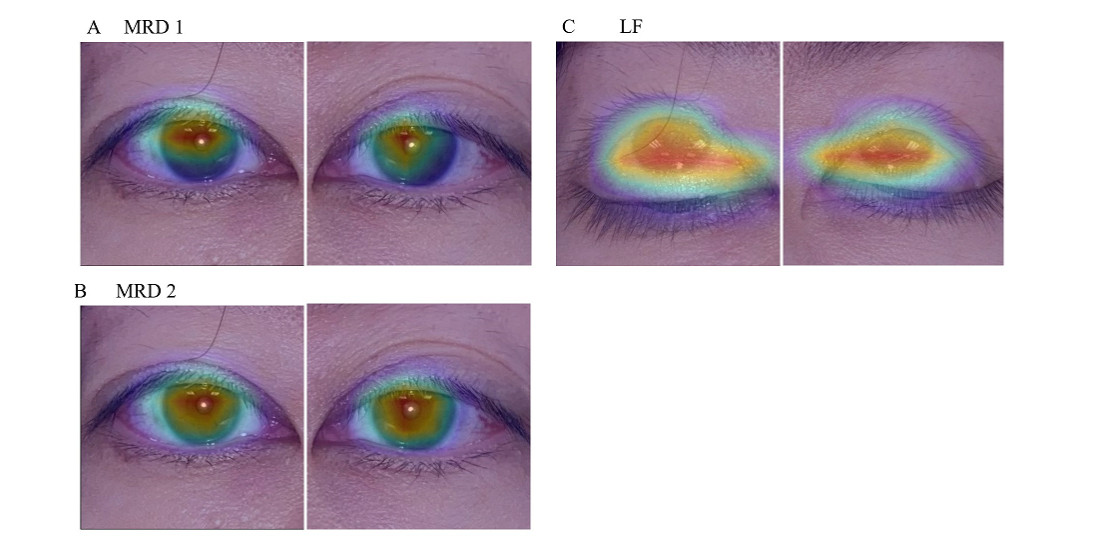
Representative heat maps of marginal reflex distance (MRD)1(A), MRD2 (B), and levator muscle function (LF) (C). The red color indicates regions with the highest discriminative value.

Representative heat maps in Figure 9 demonstrate the image region with the highest feature density and the most discriminative value (red), which was the region between the upper eyelid margin and light reflex in the MRD1 model, the region between the lower eyelid margin and light reflex in the MRD2 model, and the region between the upper eyelid margin in a merged up- and down-gaze in the LF model.

## Discussion

### Principal Findings

Since Putterman and Urist introduced the MRD, MRD1 has become an important tool for pre and postoperative ptosis evaluation [20,21]. From Putterman’s description, the MRD is measured in millimeters, and to determine MRD1, the examiner uses one hand to hold a muscle light and the other hand to hold a ruler to measure the distance from the light reflex on the cornea to the upper eyelid margin. The examiner also needs another hand (a third hand) to hold the patient’s eyebrow to prevent eyebrow elevation [22]. As a result, an examiner is less likely to perform the measurement on their own. Smartphones combine deep learning image processing as a solution to overcome this limitation.

Several automatic and semiautomatic photographic analysis approaches have been developed to provide a relatively objective assessment of MRD1 and MRD2 [4-6]. However, these studies compared their automatic and semiautomatic MRD1 and MRD2 assessments to manual measurements, not to gold-standard measurements, and the former are subjective and associated with a risk of human error. There are no automatic photographic analysis approaches for measuring the LF. To the best of our knowledge, ours is the first AI software algorithm capable of predicting MRD1, MRD2, and LF measurements with completely automated image processing and comparison of the prediction results with gold-standard measurements.

Manual MRD1, MRD2, and LF measurements are time-consuming, subjective, and have a limited precision of approximately 0.5 mm. According to Boboridis et al [2], the mean difference in measured MRD between doctors with varying degrees of experience was up to 0.5 mm, indicating poor repeatability. In this study, the correlations between the gold-standard measurements and the values predicted by the MRD1 and MRD2 models were excellent and the correlation for the values predicted by the LF model was good. The ICC results showed excellent agreement between the gold-standard measurements and the predicted values by the MRD1and MRD2 models, and substantial agreement with the values predicted by the LF models. The MAE values were 0.35 mm, 0.37 mm, and 1.06 mm for the MRD1, MRD2, and LF models, respectively, and the variance increased with length. The 95% limits of agreement were −0.94 to 0.94 mm for the MRD1 model, –0.92 to 1.03 mm for the MRD2 model, and –2.63 to 2.53 mm for the LF model. These results showed that the MRD1 and MRD2 models were equivalent and might even be better than manual measurements.

The performance of the LF model was not as excellent as that of the MRD1 and MRD2 models. One reason is that the longer the measurement, the greater the variance in the measurements. The second reason is the error during photograph normalization in the LF model. In some cases, the software algorithm could not merge the up- and down-gaze orbital photographs perfectly. The third reason is overfitting, which occurs when a model does not generalize adequately from observed data to unknown data [23]. The LF model in our study had good validation results in the training set but had limited success on the test set. An extended dataset might enhance the prediction accuracy, especially in a complex model such as the LF model used in this study [24].

Some AI models face a conundrum: their performance on the test set is good, but it is significantly lower when used in a clinical scenario. One issue is that the training data are collected under stringent conditions (such as a strictly controlled photography environment), which makes it difficult for the trained model to adjust to clinical situations (such as at the clinic). In this study, the models were created to simulate a clinical scenario. The ocular photos for model training were obtained by a smartphone to simulate the doctor checking patients’ eyelid measurements in the clinic using a handheld ruler. Therefore, we believe that our model can adapt well to clinical use.

We used a deep learning algorithm to establish three models: the MRD1, MRD2, and LF models. We intend to integrate these models into a cloud-based service available on the internet. Based on these three models, we will also develop an app software contained within a smartphone, which can work offline. In the future, the examiner can use one hand to hold a smartphone and snap six images, including bilateral primary gaze, up-gaze, and down-gaze, while holding the patient’s brow with the other. The MRD1, MRD2, and LF measurements can then be predicted by the deep learning app (Figure 1). This is a quick, objective, and convenient method for obtaining MRD1, MRD2, and LF measurements. Furthermore, the examiner can check these measurements anywhere and at any time using a smartphone, which also facilitates data collection.

### Limitations

This study had some limitations. Mascara, false lashes, obvious eyelid creases, and the lack of well-merged orbital photographs interfered with the model prediction. Negative MRD1 and MRD2 levels could not be predicted, which is another limitation. In the training/validation group, the MRD1 measurements of 25 ptotic eyelids (25/740, 3.4%) without corneal light reflex were recorded as 0 mm in this study. Surprisingly, the MRD1 model predicted negative values in 18 eyelids (18/25, 72%) of these cases, implying that the algorithm may eventually learn to predict negative values on its own. When taking orbital photographs, fine movement of patients or the smartphone cannot be completely avoided, resulting in imperfectly merged images, which is a defect of our current algorithm. The merged photos will be displayed by the cloud-based service and app software in the future, so that examiners can discard the images that are not perfectly merged and retake orbital photographs to obtain better-merged images.

### Conclusion

In this study, we developed the first smartphone-based AI-assisted image processing algorithm for eyelid measurements. MRD1, MRD2, and LF measurements can be taken in a quick, objective, and convenient manner. Furthermore, by using a smartphone, the examiner can check these measurements anywhere and at any time, which also makes data collection easier.

## Abbreviations

AI: artificial intelligence
CNN: convolutional neural network
ICC: intraclass correlation coefficient
LF: levator muscle function
MAE: mean absolute error
MAIA: medical artificial intelligence assistant
MRD: marginal reflex distance
MSE: mean square error
